# Qualitative and Quantitative Analysis of Tire Wear Particles (TWPs) in Road Dust Using a Novel Mode of Operation of TGA-GC/MS

**DOI:** 10.1021/acs.estlett.4c00937

**Published:** 2024-12-12

**Authors:** Kieran S. Evans, Daniel Baqer, Marc-Krystelle Mafina, Maya Al-Sid-Cheikh

**Affiliations:** 1 EaStCHEM School of Chemistry, University of Edinburgh, Joseph Black Building, David Brewster Rd, Edinburgh, EH9 3FJ, United Kingdom; 2 PerkinElmer, Chalfont Road, Beaconsfield, Buckinghamshire, HP9 2FX, United Kingdom; 3 School of Chemistry and Chemical Engineering, University of Surrey, Stag Hill, Guildford, GU2 7XH, United Kingdom

**Keywords:** tire wear particles, analytical chemistry, evolved gas analysis, TGA-GC/MS

## Abstract

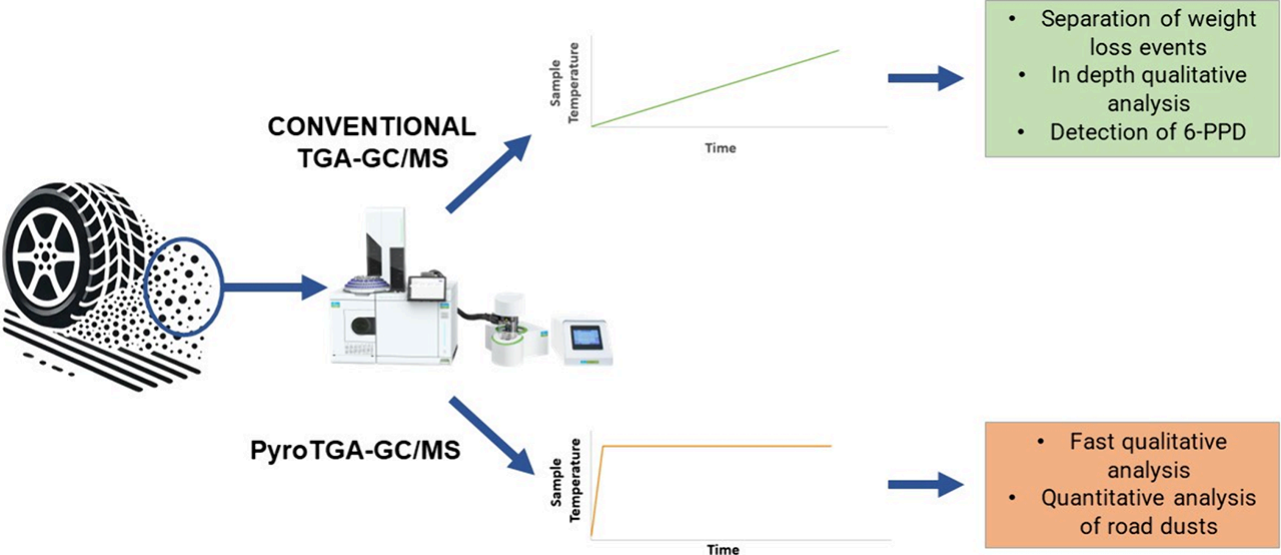

Detecting and quantifying tire wear particles (TWPs) in the environment pose a unique environmental challenge due to their chemical complexity. There are emerging concerns around TWPs due to their potential high numbers of particles released, outnumbering microplastics, as well as the leaching of toxic additives such as 6-PPD which has been linked to the death of salmon even when present at very low levels (<0.1 μg/L). Analytical techniques such as pyrolysis gas chromatography mass spectrometry (Py-GC/MS) and thermal extraction-desorption gas chromatography mass spectrometry (TED-GC/MS) have been used but also demonstrate limitations including low sample mass, low sample throughput, and complex characterization and quantification procedures. This work aims to overcome these challenges by developing a new approach which utilizes a coupling between thermogravimetric analysis (TGA) and gas chromatography–mass spectrometry (GC/MS). This work is the first to harness conventional TGA-GC/MS for the analysis of tire rubber, with the detection of additives such as 6-PPD, while also pioneering a novel mode of operation, PyroTGA-GC/MS, using fast heating to enable robust quantitative analysis of TWPs in road dust. The limits of detection and quantification of 0.08/0.16 μg and 0.20/0.40 μg for SBR and PI, respectively, are lower than those achieved using Py-GC/MS and TED-GC/MS for SBR and align with those achieved for PI. This study reveals a clear link between the ratio of PI to SBR and the proportion of heavy goods vehicles. This work solves key issues in tire particle analysis related to sample size and throughput. By overcoming these limitations, we introduce a technique that provides an economically viable solution for large-scale commercial analysis of tire rubber and particles.

## Introduction

Publicly available data on tire weights, lifespan, and abrasion-related loss reveal staggering tire wear particles (TWPs) emission in the UK. Passenger vehicles contribute up to 100,500 tonnes of TWPs annually, with heavy goods vehicles adding another 8,200 tonnes.^1−5^ The emission of TWPs poses significant health risks to humans and animals due to harmful additives, including polyaromatic hydrocarbons like benzo[a]pyrene,^6^ plasticizers like bis(2-ethylhexyl)phthalate,^7^ and antiozonants like *N*-(1,3-dimethylbutyl)-*N*′-phenyl-1,4-benzenediamine (6-PPD).^8^ Significant concern has been raised over the degradation product of 6-PPD, 6-PPD-quinone, which poses risks to aquatic life even at concentrations as low as 0.1 μg/L.^8,9^ These toxic substances can threaten ecosystems and human health, highlighting the urgent need for monitoring and mitigation measures to manage the risks of TWP emissions.^10^

TWPs and their additives present significant analytical challenges. With around 15–20 distinct rubber types on the market, and additional variability due to proprietary formulations, tires are complex composites. They typically consist of natural rubber, fillers, synthetic polymers, antioxidants, antiozonants, curing systems, steel, and textile—all tailored to enhance performance attributes like wear resistance and traction.^11^ Analytical methods for TWPs, like FT-IR and Raman microscopy, mirror those for microplastics^12,13^ but face limitations. Carbon black’s high absorbance hampers FT-IR, while Raman microscopy’s long run times slow analysis,^14,15^ highlighting the need for better detection and characterization techniques.

Thermoanalytical techniques employing gas chromatography–mass spectrometry (GC/MS) are increasingly used to detect and quantify gases or pyrolysates from thermal degradation. Pyrolysis-GC/MS (Py-GC/MS) is widely favored for its availability^16^ and detection limits, yet it suffers from low sampling mass (<1 mg), which affects repeatability in heterogeneous samples like road dusts. In contrast, thermal extraction-desorption-GC/MS (TED-GC/MS) can handle sample masses up to 100 mg and provides gravimetric data collection.^17^ However, its lengthy analysis times limit throughput to just 21 samples per week, including necessary blank runs.^18^ A promising solution to these challenges is thermogravimetric analysis coupled with gas chromatography–mass spectrometry (TGA-GC/MS), which can address the limitations of current thermoanalytical methods for TWP analysis.

TGA-GC/MS heats samples under an inert gas purge, collecting gas at key points based on mass loss. The initial scan identifies injection points, providing gravimetric data on the magnitude and temperature of each weight loss event. TGA-GC/MS enables targeted analysis of gases evolved at specific degradation points, improving the separation of tire rubber components by boiling point or degradation temperature. While it has been applied to pristine MPs and in mussel samples,^19,20^ its full potential in direct TGA-GC/MS coupling remains underexplored. Here, we pioneer the characterization and quantification of TWPs and their additives in complex environmental matrixes. Conventional TGA-GC/MS, with slow heating rates of 5–40 °C/min, fails to produce sufficient analyte gas within a 10 s injection window, limiting sensitivity and making accurate quantification challenging. Despite many studies utilizing TGA alongside Py-GC/MS, none has achieved direct coupling.

In this work, we fully harnessed the potential of TGA-GC/MS to characterize and quantify TWPs for the first time. Our method fuses both processes in one technique, enabling direct analysis and quantification of gases evolved at each stage of weight loss during the TGA experiment. This approach delivers a comprehensive understanding of a material in a single run and allows for high-throughput analyses. We present two methods using the direct coupling of TGA-GCMS. The first is the “conventional TGA-GCMS”, where we enhance the method’s flexibility to successfully detect tire additives like 6-PPD, while also addressing the limitations in TWP analysis. The current approach injects gas over a 10 s window during low mass, which can severely compromise sensitivity. To address this limitation, we pioneer “PyroTGA-GC/MS”, a new method that heats samples at rates exceeding 400 °C/min (typically 500 °C/min) and directly injects them into GC/MS. This method significantly enhances sensitivity by increasing the volume of evolved gas during the injection window, enabling accurate quantification of polymers in environmental samples. This novel technique enables analysis of larger samples (up to 100 mg), offering a more representative analysis compared to Py-GC/MS, especially where inhomogeneity is a concern. It streamlines analysis, allowing results in as little as 25 min per sample. In this work, we demonstrate how these two methods facilitate the detailed analysis of both TWPs and their additives in the environment.

## Methods and Materials

### Chemicals

Standards of styrene–butadiene rubber (SBR, Scientific Polymer Products Inc., Ontario New York), polyisoprene (PI, Sigma-Aldrich), and deuterated polystyrene (d_8_PS, Polymer Source, Dorval, Canada) were used for the creation of calibration standards.

### Environmental Samples

Environmental road dust samples (approximately 1 kg of each) were collected from the M25 motorway, A34 Newbury Bypass, and at various points along the A4 in central London (see Table S1 for details). Road dust samples were collected using a nonplastic dustpan and brush and stored in labeled paper bags. Samples were dried for 2 days at 70 °C.

### Tire Samples

Tread and sidewall samples were taken from four tire samples (detailed in Table S2). 5–10 mg portions of rubber were cut from each sample for TGA-GC/MS analysis with no further sample preparation required. Tire samples were measured to investigate the composition and thermal degradation of tire tread and sidewall. For each sample, a survey scan consisting of a standalone TGA measurement was first used to determine GC/MS injection points. Samples were then measured with injection to the GC/MS triggered at the appropriate time using an electrical contact closure. In total, 8 samples of different tire rubber formulations were measured in order to determine suitable marker compounds to use in later analysis in conjunction with analysis of pure elastomer standards, in line with the number used in similar studies.^21^

### GC/MS Parameters

In both modes, the transfer line and GC/MS parameters remained unchanged. Evolved gases were pulled through a TL9000e (PerkinElmer, Shelton, CT) and heated to 260 °C at 80 mL/min. A 100 μL sample was analyzed by using a gas chromatograph (Clarus 690, PerkinElmer, Shelton, CT) equipped with an Elite-1MS column. The column was programmed with a temperature ramp: 40–100 °C at 5 °C/min, 100 to 300 °C at 10 °C/min, followed by 5 min isothermal at 300 °C, 12.2 psi He carrier gas. Eluted compounds were detected using a mass spectrometer (SQ8T, PerkinElmer, Shelton, CT) with the following settings: GC/MS interface at 300 °C, ion source temperature at 250 °C, 0.5 min delay, interscan delay of 0.02 s, electron ionization at 70 eV, and a mass range of 50–350 *m*/*z*. For quantitative analysis, single ion monitoring (SIM) scans targeted masses 54, 112, and 121 to measure 4-vinylcyclohexene (4-VCH), deuterated styrene, and limonene, respectively.

For analysis of the first injection in conventional TGA-GC/MS analysis, the GC program was optimized for the detection of 6-PPD (10 min isothermal at 30 °C, 30 to 90 °C at 5 °C/min, 90 to 300 °C at 20 °C/min, 5 min isothermal at 300 °C).

This method offers the advantage of using the same hardware for both conventional and PyroTGA-GC/MS, with the difference lying solely in how TGA is operated.

### Conventional TGA Parameters

Samples were heated in the TGA from 50 to 800 °C at 10 °C/min under a nitrogen purge (100 mL/min) followed by heating from 800 to 1000 °C at 10 °C/min under an air purge (100 mL/min).

### PyroTGA Parameters

The TGA furnace was preheated to 600 °C with a nitrogen purge (250 mL/min) before being raised onto the sample. The sample was pyrolyzed for 18 s before GC/MS analysis.

Although TGA-GC/MS can handle up to 100 mg of analogues, the optimal sample size was tailored for maximum analytical precision. For the standards, 1 mg of pure polymer was analyzed to identify the thermal degradation products. For environmental samples, 15–20 mg was used. d_8_PS served as the internal standard for quantification. Polybutadiene, although present in tire rubber, was excluded from the quantitative study due to the nonspecific nature of its pyrolysis products, as demonstrated by Eisentraut et al.^21^ Key degradation products of pure SBR and PI are listed in Table S3 and shown in Figure S1. A major advantage of the gravimetric component of this technique is its ability to confirm complete decomposition by monitoring weight loss.

### Standards

Standards of SBR, PI, and d_8_PS were dissolved in THF (HPLC grade, Sigma-Aldrich) to prepare concentrations of 10 mg/mL, 100 mg/mL, and 50 mg/mL. For calibration, aliquots of standards were added to a 50 μL TGA crucible containing ca. 20 mg of pyrolyzed road dust as a simulated matrix. A 10 μL (500 μg) spike of deuterated polystyrene was also included as an internal standard, following the method established by Unice et al.^22^ THF was used to dissolve the calibration and internal standards, which were then added to TGA pans at precise concentrations. The standards were dried before analysis to remove THF, leaving only the polymer. Procedural blanks were tested to evaluate any potential impacts from THF as a solvent (Figure S2).

### Limit of Detection (LOD) and Quantification (LOQ)

The LOD and LOQ were calculated using the quintuple signal-to-noise ratio for LOD and ten times the signal-to-noise ratio for LOQ, as per Gimeno et al.^23^ Unlike traditional methods, the LOD is expressed as an absolute weight rather than a concentration. Using this method, the LOD values for SBR and PI were 0.08 μg and 0.20 μg, respectively, while LOQs were found to be 0.16 μg for SBR and 0.40 μg for PI. These results demonstrate superior sensitivity compared to detection limits achieved using Py-GC/MS (1–2 μg) and TED-GC/MS (approximately 0.2 μg).^21,24^

## Results and Discussion

### Analysis of Tire Rubber Using Conventional TGA-GC/MS

**Figure 1:**
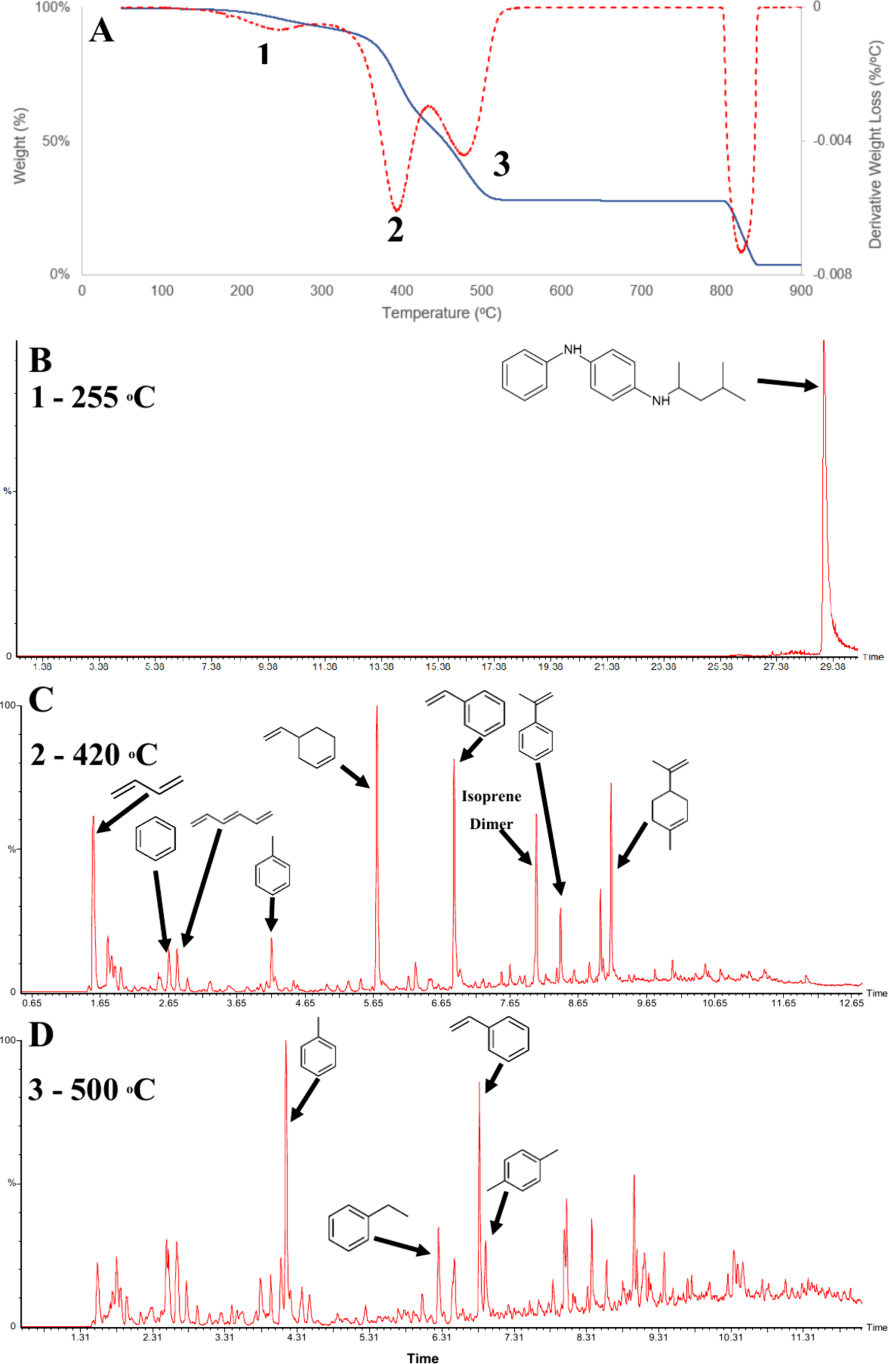
Example of (A) weight loss (blue) and derivative weight loss (red) TGA curves of tire rubber. (B) Chromatogram of gas evolved at 255 °C. (C) Chromatogram of gas evolved at 420 °C. (D) Chromatogram of gas evolved at 500 °C. Each sample was measured repeatedly (*n* = 3) for accuracy.

TGA-GC/MS simultaneously elucidates the thermal behavior and composition for tire tread and sidewall, facilitating the identification and quantification of marker compounds in environmental samples. Figure 1(A) presents the TGA curve of a tire tread, revealing three distinct weight loss events at 255, 420, and 500 °C. The evolved gases from these events were injected into the GC to identify the specific compounds generated during degradation.

The first weight loss (up to 255 °C, 4.5%) likely corresponds to the volatilization of volatile components such as additives. The single ion chromatogram in Figure 1(B) shows mass 211, the dominant ion in the 6-PPD mass spectrum, confirming its presence at RT 29.04 min. This suggests that, during gradual heating of tire rubber, 6-PPD volatilizes without degradation. Additional compounds identified in the total ion chromatogram include sulfur dioxide (RT 1.55 min) and cyclohexanethiol (RT 16.62 min). The detection of 6-PPD was validated by comparing its mass spectra with a standard run under identical conditions (Figure S3).

The second (420 °C, 37.1%) and third (500 °C, 27.4%) weight losses reveal a two-step decomposition of tire elastomers, producing varying concentrations of key degradation markers (Figure 1C and D). This distinction is critical for identifying reliable markers for quantification. Selecting markers requires a balance between selectivity and sensitivity. For instance, while styrene is highly sensitive to SBR concentration, its selectivity is limited due to overlap with plastic pollutants like polystyrene. Therefore, 4-VCH, a known SBR degradation product, was selected for its superior selectivity and adequate sensitivity compared to styrene.^25^

A similar challenge arose for PI. While isoprene was highly abundant, it likely originates from multiple organic sources,^26^ and its poor retention on single-phase analytical columns caused overlap with species like butadiene. Limonene, though less abundant, offered greater selectivity and is a known PI degradation product, formed through a spontaneous Diels–Alder reaction between isoprene radical fragments during pyrolysis.^27^ Thus, limonene was selected as the marker for PI quantification due to its selectivity and superior GC resolution. Specificity of the chosen markers was confirmed through PyroTGA-GC/MS analysis of common polymers, where neither 4-VCH nor limonene was detected. Major degradation products from these polymers are listed in Table S4. To confirm no interference from limonene derived from natural sources, a sample containing natural materials (confirmed by visible microscopy) but no PI (confirmed by the lack of marker compounds) was measured, showing no limonene response (Figure S4).

TGA data provide additional insights into the material, such as determining the residual carbon content from tire rubber pyrolysis. By switching the purge gas to air at 800 °C, the sample in Figure 1 shows a residual carbon content of 24.1%. The inorganic filler content is quantified by measuring the remaining weight after heating in air, which for this sample is 3.8%.

The final step in conventional TGA-GC/MS analysis was determining the optimal temperature for the pyrolysis of road dust samples. The TGA curve in Figure 1 shows no further pyrolysis beyond 600 °C, making this the chosen temperature for the analysis.

### Determination of SBR and PI Content in Road Dust Samples

#### Calibration

Calibration for styrene–butadiene rubber and PI was established using the peak area ratios of styrene and deuterated styrene, as well as dipentene and deuterated styrene, respectively (Figure S5). Before final calibration, covariance between SBR and PI concentration was investigated by plotting the components against one another, yielding an *R*^2^ value of 0.0001, confirming their independence. The study also revealed that SBR and PI concentrations fell into distinct ranges, with SBR ranging from 10–300 μg and PI from 200–2200 μg. Quantification was based on the absolute weight, allowing flexibility in sample weights. Calibration curves for both SBR and PI showed *R*^2^ values of 0.99.

#### Validation

To validate the method’s accuracy and repeatability, samples spiked with 80 μg of SBR and 800 μg of PI were analyzed, yielding spike recoveries of 106% for SBR and 93% for PI. The relative standard deviations (RSDs) were 11.5% and 12.6% (*n* = 10), respectively, both well below the 20% threshold typically required for chromatographic methods in environmental analysis.^28^

#### Measurement of Road Dust

**Figure 2:**
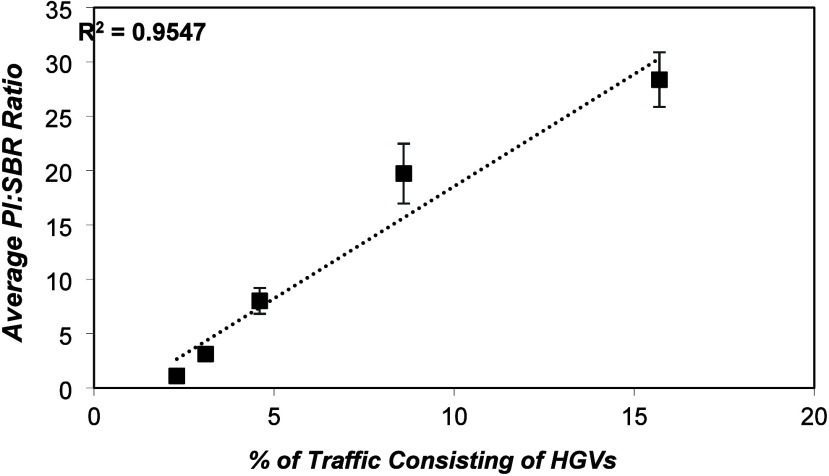
Average PI:SBR ratio in road dusts against the proportion of traffic consisting of HGVs (*n* = 21 in total, error bars = ±2σ).

Figure 2 illustrates the average PI-to-SBR ratio plotted against the percentage of heavy goods vehicle (HGV) traffic. Measurements were conducted in triplicate for three inner-city locations and sextuplicate for two highway sites, with the average SBR and PI concentrations for each location provided in Table S1. The results indicate a linear increase in PI levels in road dust as HGV traffic increases from 2.3 to 15.7%, with a strong correlation (*R*^2^ = 0.95). Notably, HGV tires contain approximately twice as much natural rubber and half the synthetic rubber compared to passenger vehicle tires.^29^ The higher natural rubber content in HGV tires stems from its superior physical properties, making it more suitable for heavy-duty applications.^30^ Consequently, roads with a higher proportion of HGV are expected to exhibit elevated levels of PI relative to SBR in road dust. Future work will expand the sampling area to further investigate and validate this relationship.

## Environmental Implications

This work highlights two TGA-GC/MS modes for analyzing complex environmental samples, like TWPs and their additives, with conventional TGA-GC/MS pinpointing degradation markers for further study. It also optimizes analysis by revealing pyrolysis behavior. PyroTGA-GC/MS generates bulk gas for chromatography, boosting sensitivity and offering a more representative analysis of heterogeneous samples. This technique is ideal for complex analytes like TWPs in challenging matrixes such as road dust and sediments. Linking elastomer ratios to traffic demographics could inform targeted mitigation strategies.

PyroTGA-GC/MS enables rapid quantification of TWPs in under 25 min with minimal sample preparation, accommodating large sample sizes (up to 100 mg). In addition to its analytical versatility, PyroTGA-GC/MS provides critical insights into TWP degradation and additives, achieving limits of detection of 0.08 and 0.20 μg for SBR and PI, respectively. This sensitivity makes it suitable for challenging matrixes with predicted environmental concentrations of TWPs averaging 30 mg/L and 120 μg/L in runoff and surface waters, respectively, as well as sampling heterogeneous environments such as sediments.^31,32^ Its speed and efficiency support high-throughput environmental monitoring, crucial for assessing the impact of regulations, not only evaluating the effectiveness of such policies but also ensuring that unintended environmental consequences, such as overlooked pollutant sources, are detected and addressed promptly.

## Supporting Information Available

Additional data and detailed analyses to complement the main findings. Table S1 lists the GPS coordinates of road dust samples analyzed, ensuring spatial context for the quantified results. Table S2 summarizes tire properties measured via TGA-GC/MS to identify common elastomer markers. Tables S3 and S4 outline major degradation products of styrene–butadiene rubber (SBR), polyisoprene (PI), and other polymers as detected by PyroTGA-GC/MS. The figures further illustrate the experimental findings. Figure S1 presents a representative chromatogram from the M25 road dust, showcasing key pyrolysis products. Figure S2 demonstrates the procedural blank test, confirming minimal interference from the solvent. Figure S3 compares the mass spectra of 6-PPD with a tire sidewall to confirm identification. Figure S4 highlights limonene responses to differentiate polyisoprene-containing samples from natural materials. Figure S5 provides calibration curves for SBR and PI, underscoring the method’s quantitative robustness (PDF)
