# Supplementary material for: Qualitative and Quantitative Analysis of Tire Wear Particles (TWPs) in Road Dust Using a Novel Mode of Operation of TGA-GC/MS
Source: Environ Sci Technol Lett. 2024 Dec 12;12(1):79–84. doi: 10.1021/acs.estlett.4c00937 (PMC11736838; doi:10.1021/acs.estlett.4c00937)
Supplement: Supplementary file 1 — ez4c00937_si_001.pdf [file ez4c00937_si_001.pdf]

Supporting Information - Qualitative and Quantitative Analysis of Tyre Wear Particles (TWPs) in Road Dust Using a Novel Mode of Operation of TGA-GC/MS

Kieran S. Evans[1,2], Daniel Baqer[3], Marc-Krystelle Mafina[2], Maya Al-Sid-Cheikh[1]*

1. School of Chemistry, University of Edinburgh, Joseph Black Building, David Brewster Rd, Edinburgh, United Kingdom
2. PerkinElmer, Chalfont Road, Beaconsfield, Buckinghamshire, United Kingdom
3. School of Chemistry and Chemical Engineering, University of Surrey, Stag Hill, Guildford, United Kingdom

Tables

Table S1. GPS Coordinates of Quantified Road Dust Samples. 2
Table S2. Information about tyres measured by TGA-GC/MS to determine common markers found from elastomers. 2
Table S3. Major degradation products of styrene-butadiene rubber and polyisoprene. 3
Table S4. Major degradation products of common polymers measured by PyroTGA-GC/MS. 4

Figures

Figure S1. Example chromatogram from the initial qualitative PyroTGA-GC/MS measurement of a road dust from the M25 (left to right – Isoprene, 4-Vinylcyclohexene, Styrene, α-methylstyrene, Limonene, But-3-en-1-ylbenzene). 5
Figure S2. Results from procedural blank after addition and evaporation of THF (red = 54 m/z, green = 112 m/z, purple = 121 m/z). No interference seen aside from unretained peak at 1.50 min. 5
Figure S3. Mass spectra obtained after injection at 255 °C at 29.04 minutes for a standard of 6-PPD (top, green) and a tyre sidewall (bottom, red). 6
Figure S4. Limonene response for a sample that contains polyisoprene (red) and a sample that contains natural material but no polyisoprene (green). 6
Figure S5. Calibration curves for styrene-butadiene (top) and polyisoprene (bottom). 7

Table S1. GPS Coordinates of Quantified Road Dust Samples.

Sample	Coordinates	Average SBR Concentration (mg/g)	Average PI Concentration (mg/g)	Collection Date
**M25**	(51.269025, -0.009191)	0.33	6.59	15th June 2021
**A34**	(51.419583, -1.345944)<br>(51.419583, -1.345944)<br>(51.419666, -1.346275)<br>(51.419666, -1.346275)	0.42	11.70	15th June 2021
**Central London (A4)**	(51.495392, -0.179095)	5.44	16.95	29th October 2009
	(51.501978, -0.151322)	12.54	13.72	
	(51.510865, -0.129062)	1.61	12.78	

Table S2. Information about tyres measured by TGA-GC/MS to determine common markers found from elastomers.

Tyre Sample	Type	Tyre Part	Ash Content (%)
1	New (All Weather)	Tread	40.1
		Sidewall	3.8
2	Used (All Weather)	Tread	19.4
		Sidewall	2.6
3	New (All Weather)	Tread	33.8
		Sidewall	3.3
4	New (Winter)	Tread	31.0
		Sidewall	2.6

Table S3. Major degradation products of styrene-butadiene rubber and polyisoprene.

SBR			PI		
Compound Name	Structure	Identifier Ions	Compound Name	Structure	Identifier Ions
Styrene	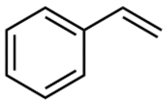	104, 78, 51	Isoprene	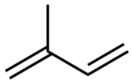	53, 68, 67
α-methyl Styrene	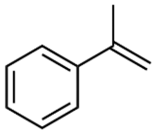	118, 103, 78	Limonene	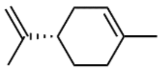	67, 68, 79, 93, 121, 136
4-Vinyl Cyclohexene	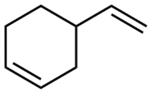	54, 79, 91, 108	Other Polyisoprene Dimers	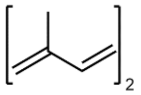	67, 68, 79, 93, 121, 137
But-3-en-1-ylbenzene	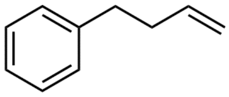	132, 91	Polyisoprene Trimers	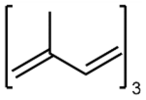	121, 107, 93, 81, 189, 204

Table S4. Major degradation products of common polymers measured by PyroTGA-GC/MS.

**Polymer**	**Degradation Product**	**Major Mass Ions (*m/z*)**
Polystyrene	Benzene	78, 51
	Toluene	92, 91, 65
	Ethylbenzene	106, 91
	Styrene	104, 103, 78, 77
	α-methylstyrene	118, 117, 103, 91, 78
	Styrene dimer	208, 130, 115, 104, 91
Polyethylene*	1-Decene (C10)	83, 70, 69, 56, 55 <u>140</u>
	1-Undecene (C11)	83, 70, 69, 56, 55 <u>154</u>
	1-Dodecene (C12)	83, 70, 69, 56, 55 <u>168</u>
	1-Tetradecene (C14)	83, 70, 69, 56, 55 <u>196</u>
Polypropylene	2,4-Dimethyl-1-heptene	126, 83, 70, 55
	3,7-Dimethyl-1-octene	83, 70, 55
	4,6,8-Trimethyl-1-nonene	85, 71, 57
Poly(ethylene terephthalate)	Vinyl benzoate	105, 77, 51
	Benzoic acid	122, 105, 77, 51
	Ethylene dibenzoate	227, 105, 77
	Divinyl terephthalate	175, 132, 104, 76

*Polyethylene produces a wide range of terminal diene, terminal monoene and saturated carbon chains (C4-C30 was observed). The fragmentation of these chains is similar and as such, the mass ion for the pyrolysis products shown in the table is shown as underlined.

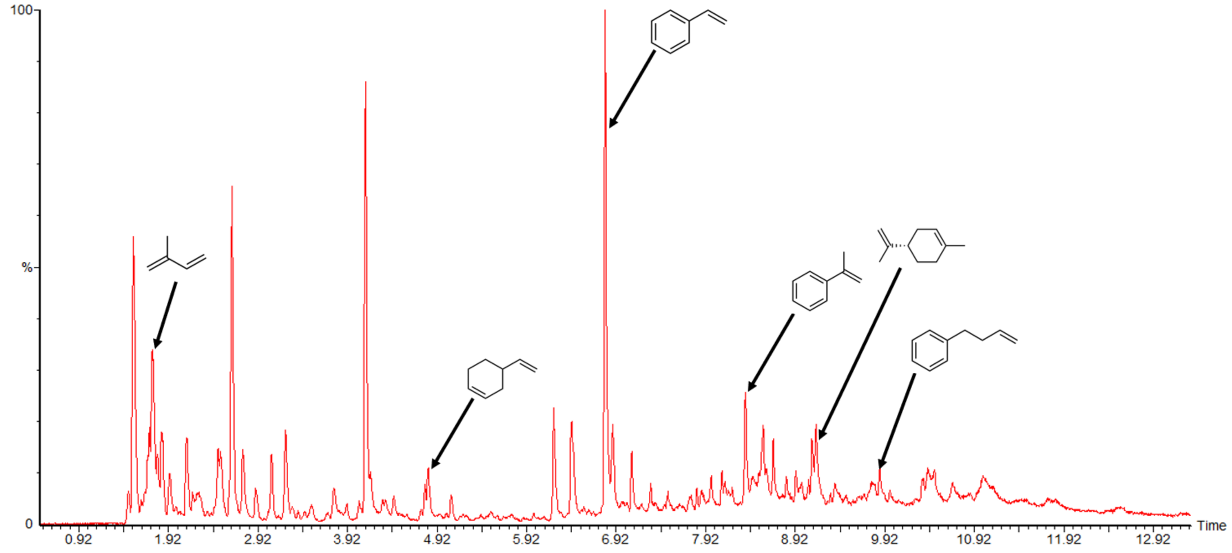

Figure S1. Example chromatogram from the initial qualitative PyroTGA-GC/MS measurement of a road dust from the M25 (left to right – Isoprene, 4-Vinylcyclohexene, Styrene, α-methylstyrene, Limonene, But-3-en-1-ylbenzene).

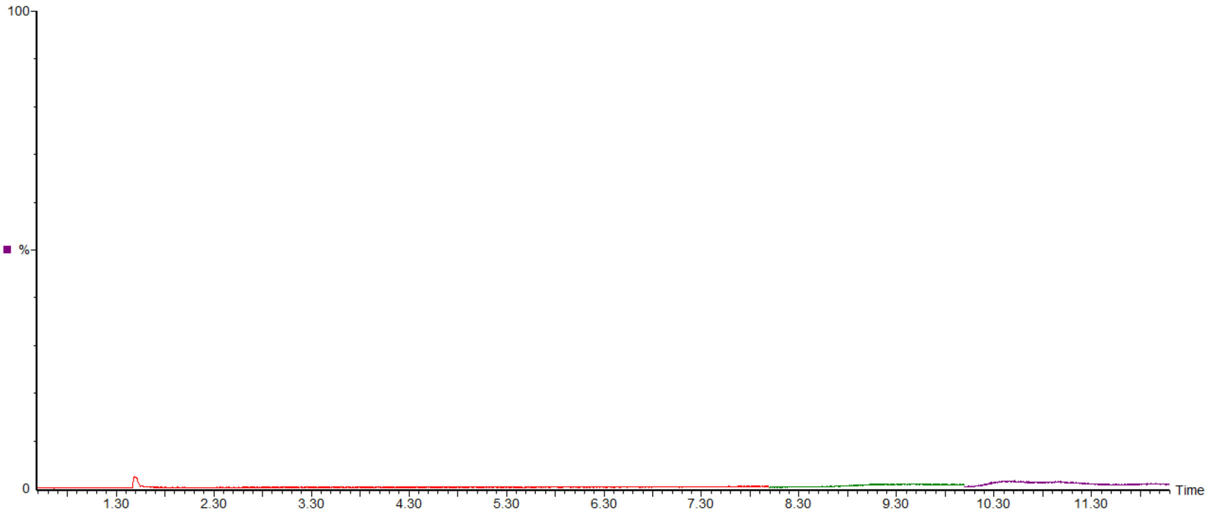

Figure S2. Results from procedural blank after addition and evaporation of THF (red = 54 m/z, green = 112 m/z, purple = 121 m/z). No interference seen aside from unretained peak at 1.50 min.

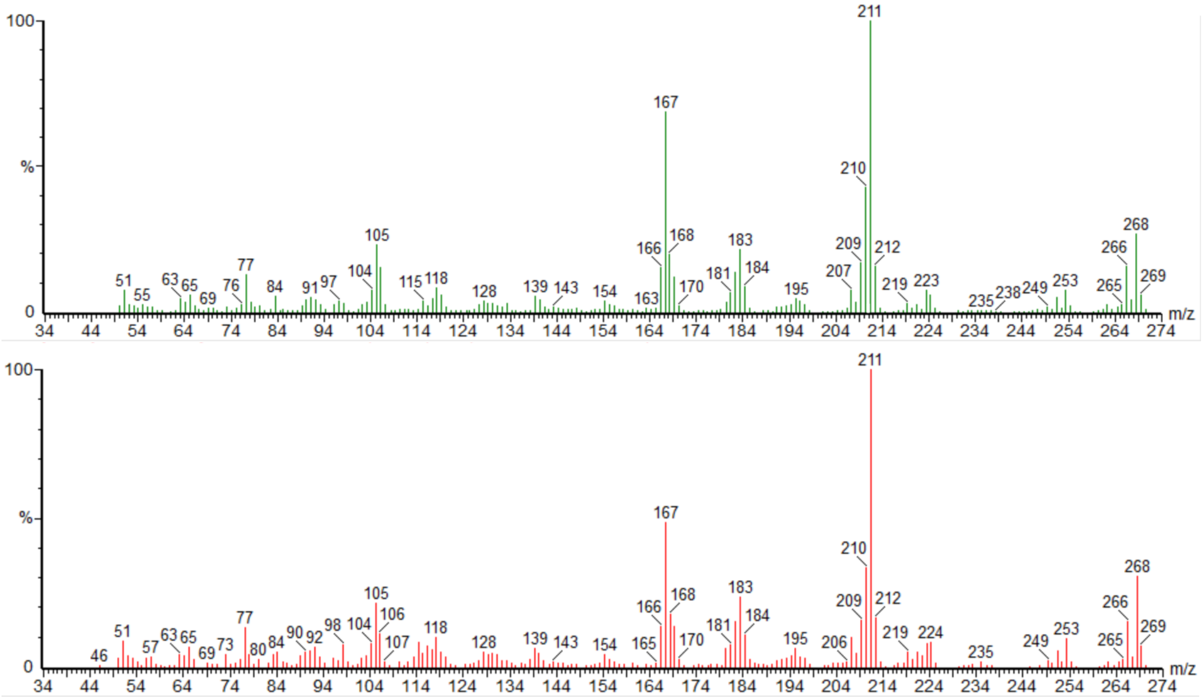

Figure S3. Mass spectra obtained after injection at 255 ^{o}C at 29.04 minutes for a standard of 6-PPD (top, green) and a tyre sidewall (bottom, red).

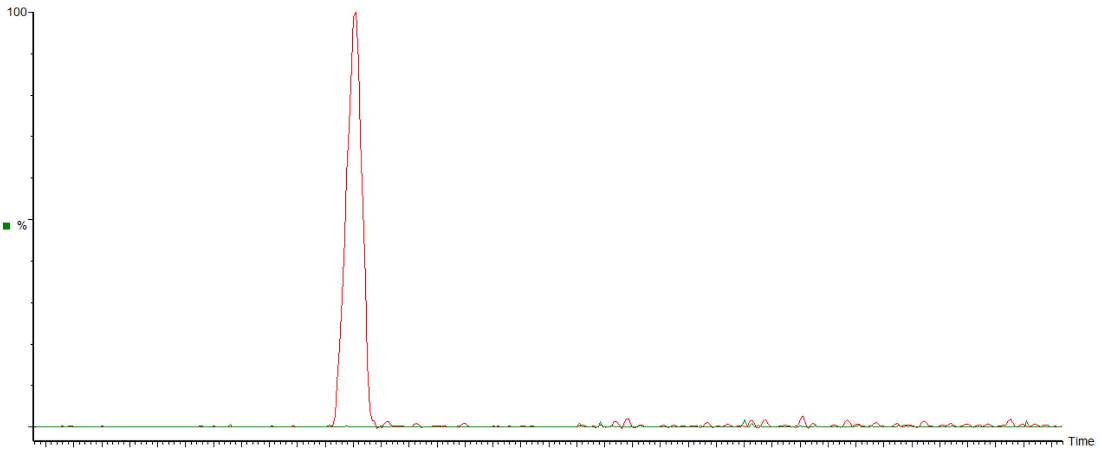

Figure S4. Limonene response for a sample that contains polyisoprene (red) and a sample that contains natural material but no polyisoprene (green).

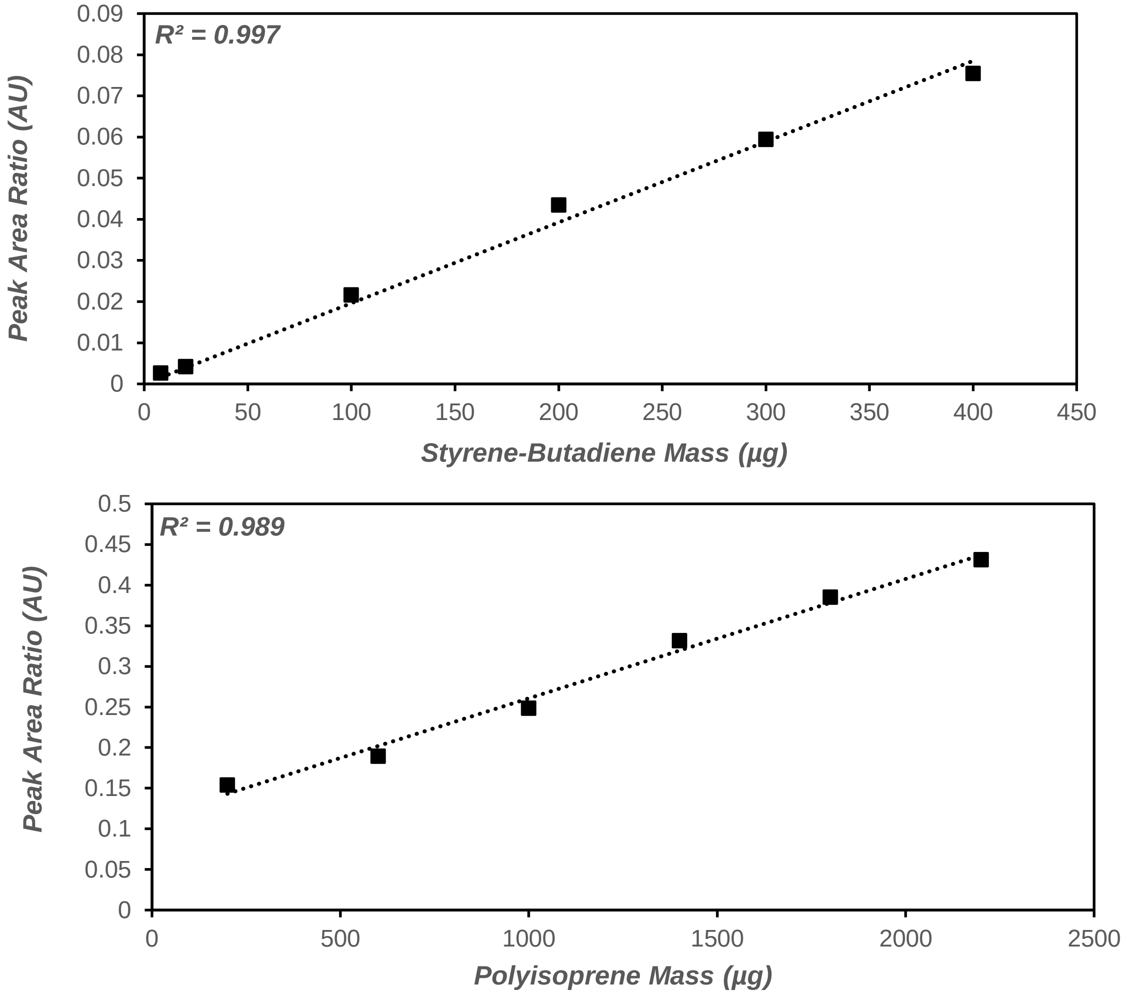

Figure S5. Calibration curves for styrene-butadiene (top) and polyisoprene (bottom).
